# Supplementary figures and images for: Suppression of interferon signaling via small-molecule modulation of TFAM
Source: eLife. 2026 Feb 6;14:RP108742. doi: 10.7554/eLife.108742 (PMC12880803; doi:10.7554/eLife.108742)

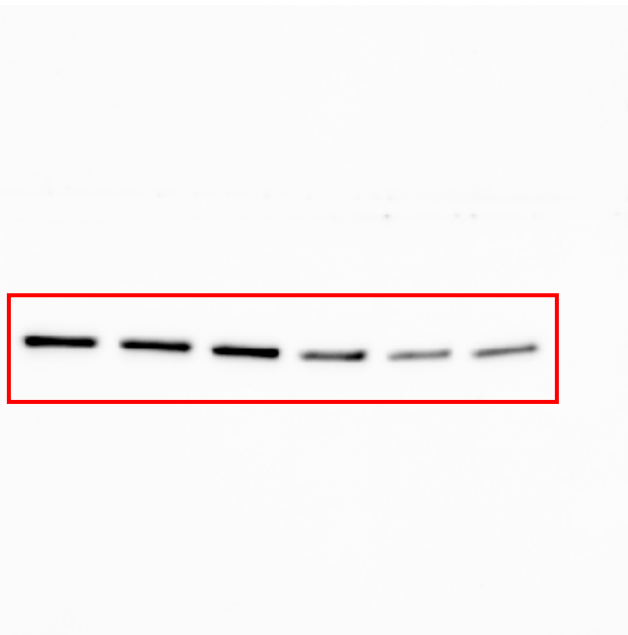

α-SMA

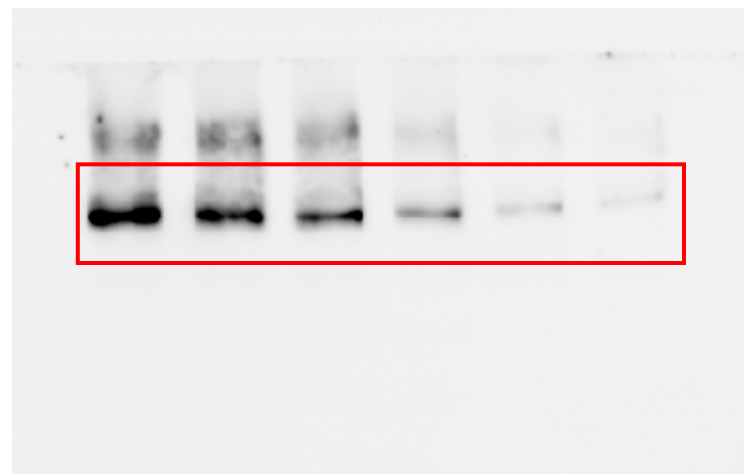

Fibronectin

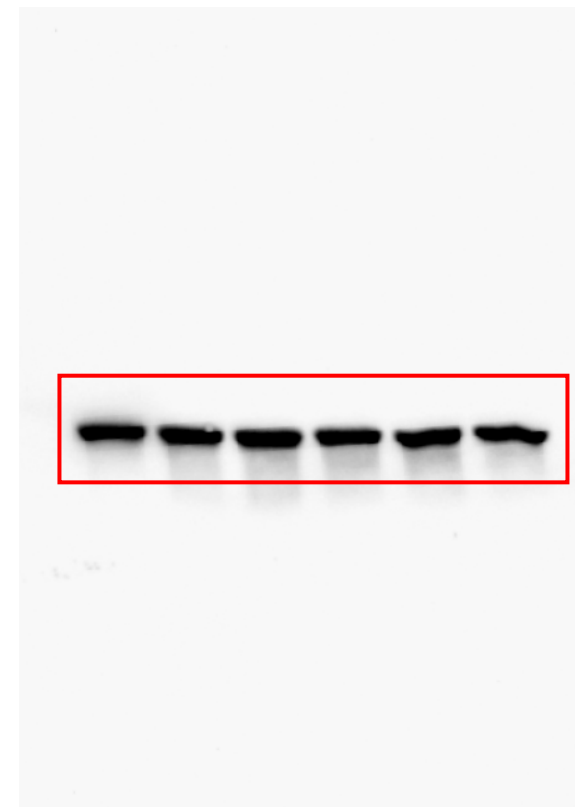

Actin

Supplement: Figure 4—source data 1. [file elife-108742-fig4-data1.pdf]

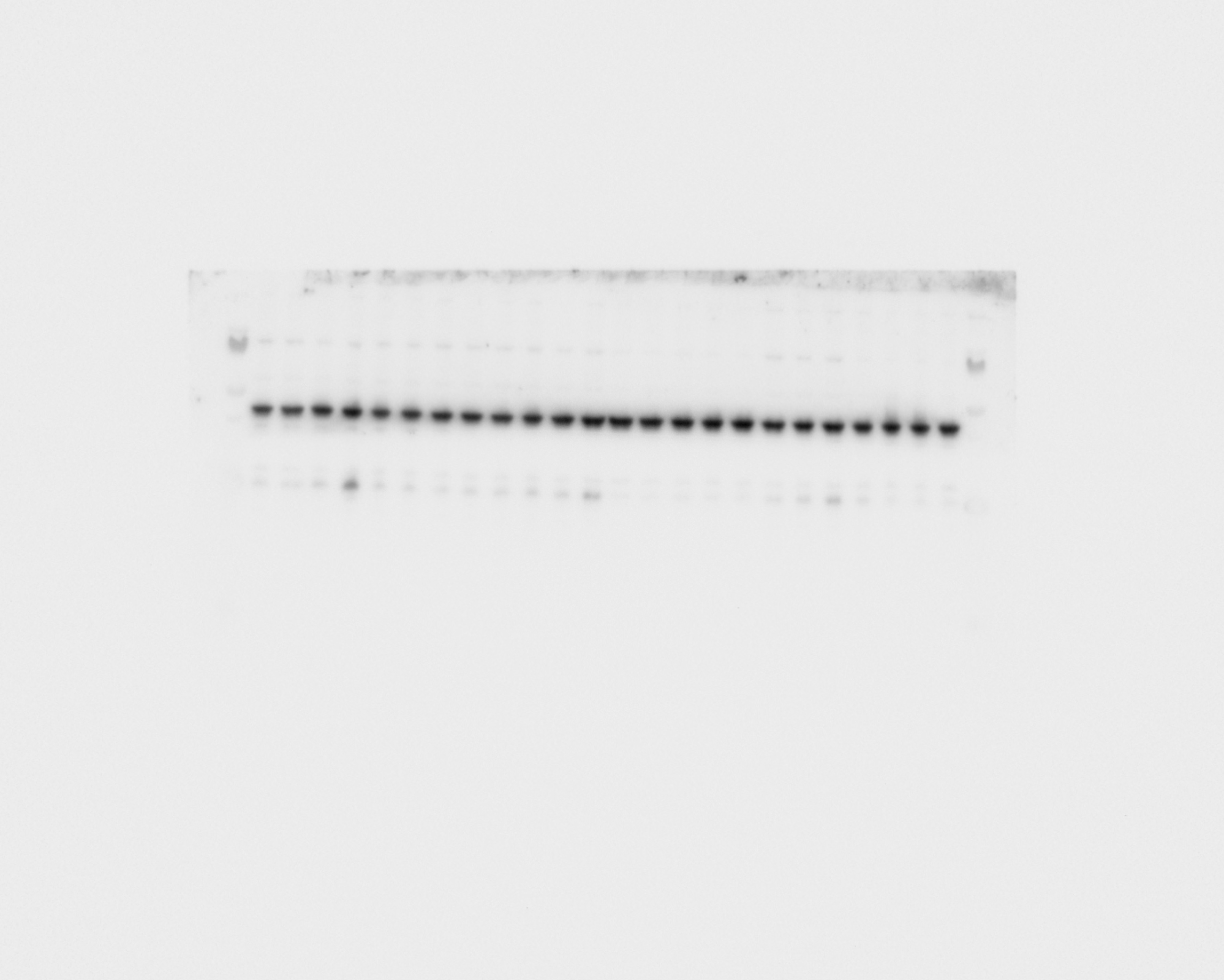

Supplement: Figure 4—source data 2. [file elife-108742-fig4-data2.zip › Figure 4-source data 1/Fig4B-1.jpg]

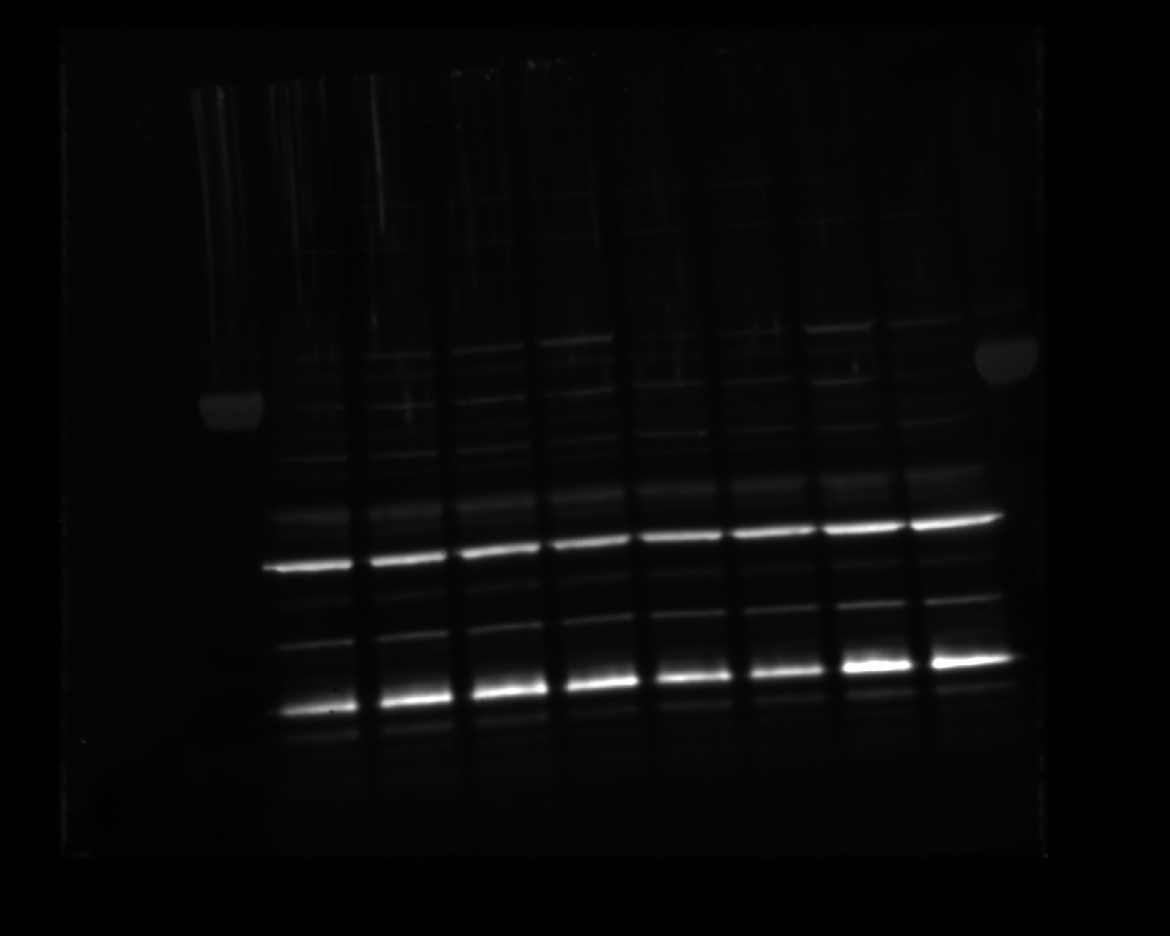

Supplement: Figure 4—source data 2. [file elife-108742-fig4-data2.zip › Figure 4-source data 1/Fig4B-2.jpg]

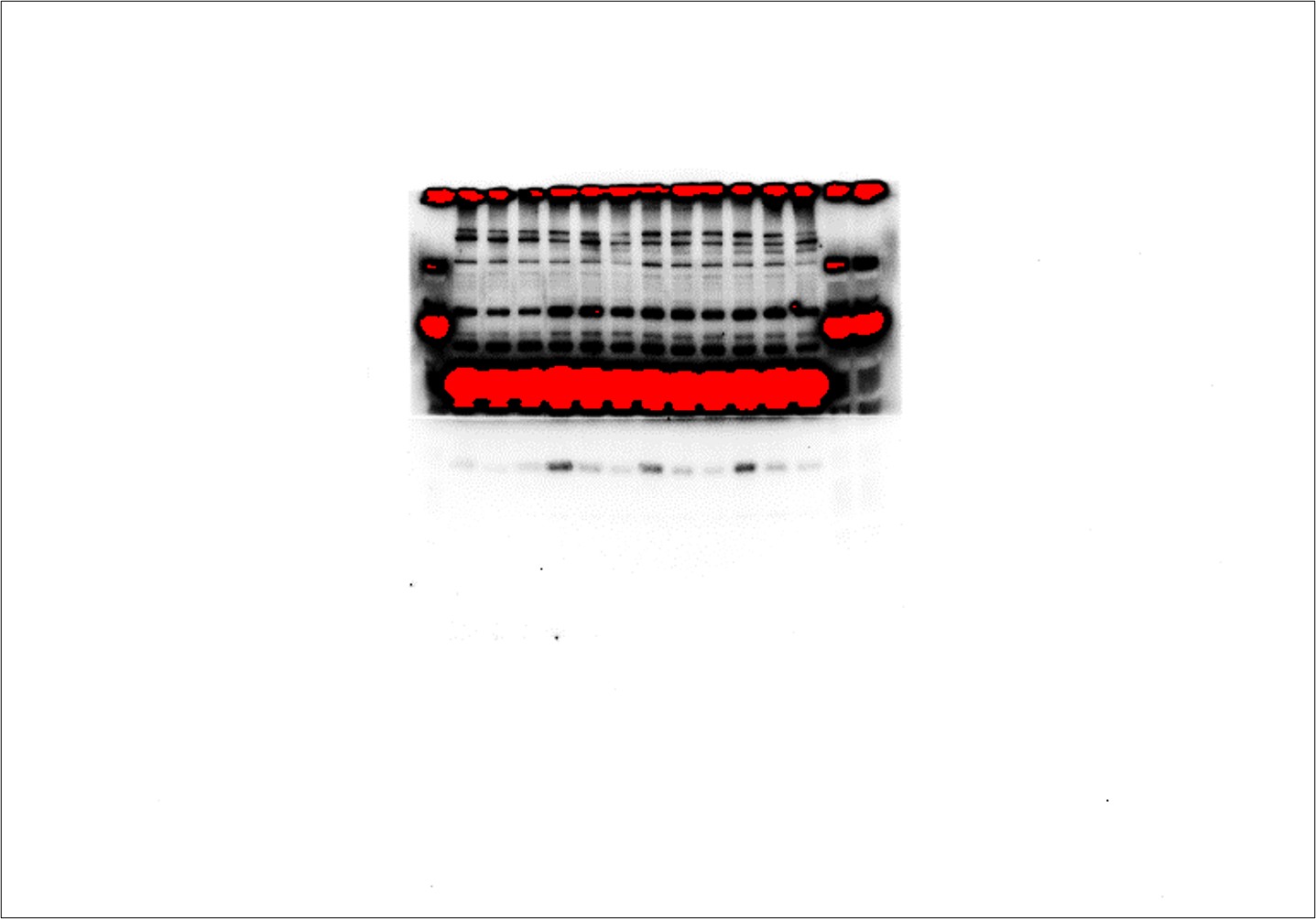

Supplement: Figure 4—source data 2. [file elife-108742-fig4-data2.zip › Figure 4-source data 1/Fig4D-1.jpg]

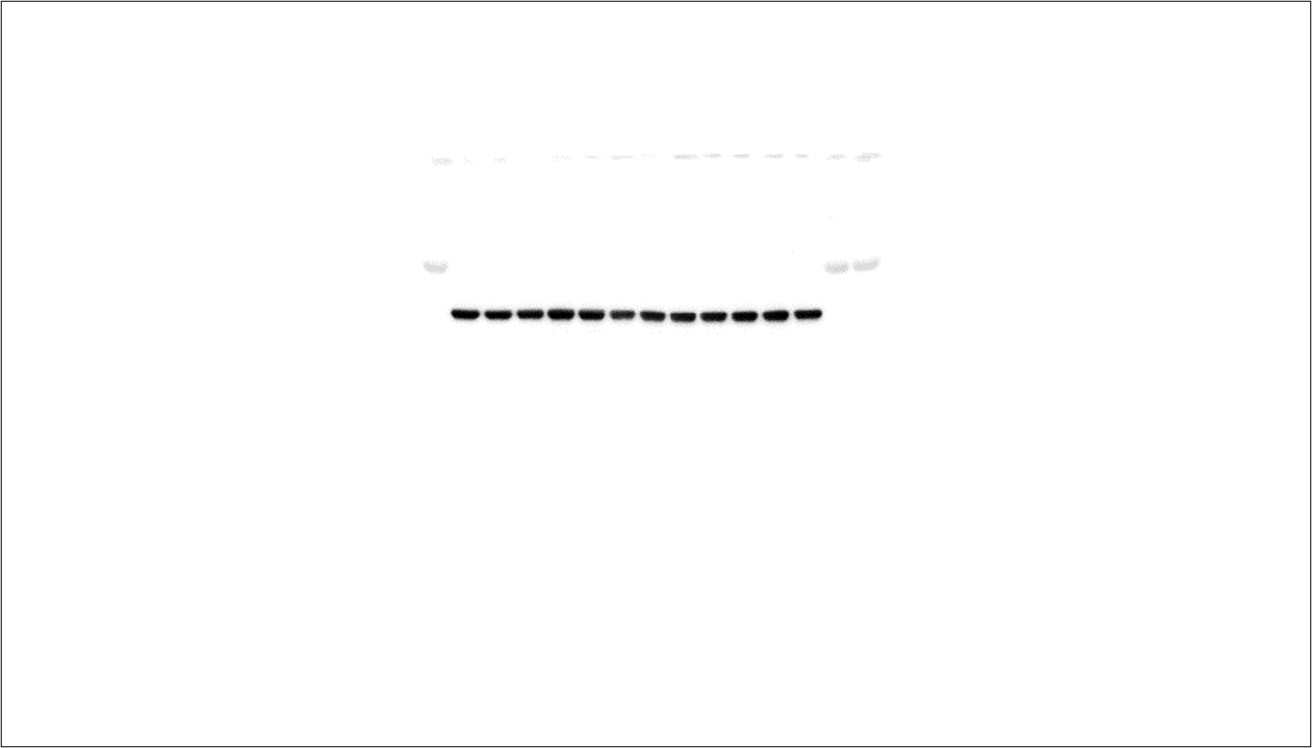

Supplement: Figure 4—source data 2. [file elife-108742-fig4-data2.zip › Figure 4-source data 1/Fig4D-2.jpg]

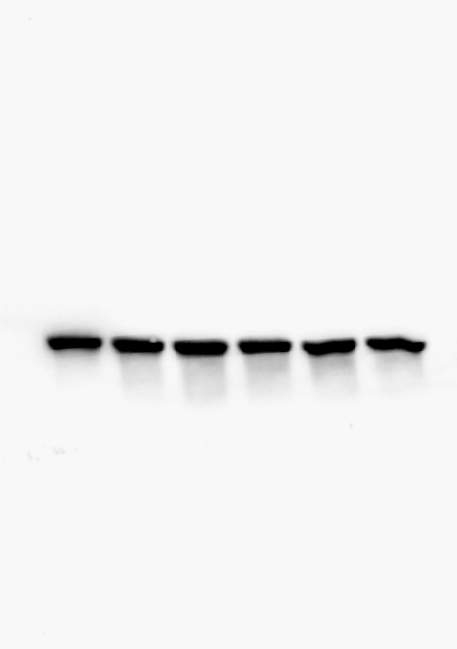

Supplement: Figure 5—source data 1. [file elife-108742-fig5-data1.zip › Figure 5-source data 1/Fig5B-ACTIN.tif]

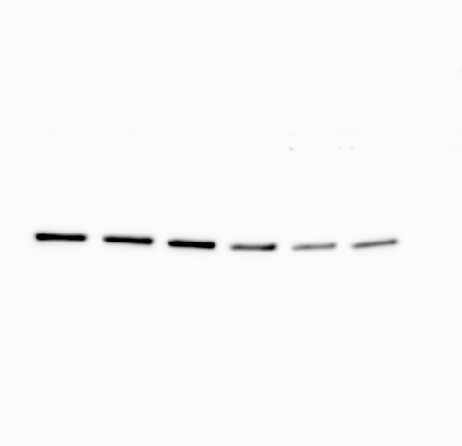

Supplement: Figure 5—source data 1. [file elife-108742-fig5-data1.zip › Figure 5-source data 1/Fig5B-ALPHA-SMA.tif]

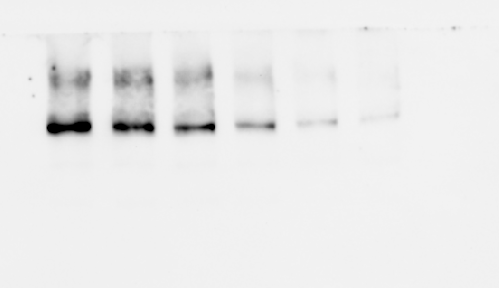

Supplement: Figure 5—source data 1. [file elife-108742-fig5-data1.zip › Figure 5-source data 1/Fig5B-FIBRONECTIN.tif]
